# Transforming Intracerebral Hemorrhage Care with Artificial Intelligence: Opportunities, Challenges, and Future Directions

**DOI:** 10.3390/diagnostics16050752

**Published:** 2026-03-03

**Authors:** Qian Gao, Yujia Jin, Yuxuan Sun, Meng Jin, Lili Tang, Yuxiao Chen, Yutong She, Meng Li

**Affiliations:** 1 Department of Neurology, Affiliated Hangzhou First People’s Hospital School of Medicine, Westlake University, Hangzhou 310006, China; 3240102624@zju.edu.cn (Q.G.); 12318500@zju.edu.cn (Y.J.); 3240106018@zju.edu.cn (Y.S.); 22318453@zju.edu.cn (M.J.); 22318465@zju.edu.cn (L.T.); 22518393@zju.edu.cn (Y.C.); 3220105987@zju.edu.cn (Y.S.); 2 Department of Neurology, Zhejiang University School of Medicine, Hangzhou 310058, China

**Keywords:** intracerebral hemorrhage, artificial intelligence, machine learning, neuroimaging, brain–computer interfaces, prognostication

## Abstract

Spontaneous intracerebral hemorrhage (ICH) is associated with substantial mortality and morbidity. Current management paradigms rely heavily on the rapid interpretation of neuroimaging and clinical data, yet are frequently constrained by limitations in processing speed, diagnostic accuracy, and prognostic precision. Artificial intelligence (AI), specifically machine learning (ML) and deep learning (DL), offers transformative potential to circumvent these challenges across the entire continuum of ICH care. This comprehensive review synthesizes the rapidly evolving landscape of AI applications in ICH management. Through a systematic evaluation of recent literature, we examine studies focused on the development, validation, or critical appraisal of AI-driven technologies for ICH care. Our analysis encompasses automated neuroimaging, computer-assisted surgical navigation, brain–computer interfaces (BCIs), prognostic modeling, and fundamental research into disease mechanisms. AI has demonstrated performance comparable to that of clinical experts in automating hematoma segmentation, predicting complications such as hematoma expansion, and refining surgical planning via augmented reality. Furthermore, BCIs present innovative therapeutic avenues for motor rehabilitation. However, the translation of these technological advances into routine clinical practice is impeded by substantial challenges, including data heterogeneity, model opacity (“black-box” issues), workflow integration barriers, regulatory ambiguities, and ethical concerns surrounding accountability and algorithmic bias. The integration of AI into ICH care signifies a paradigm shift from standardized treatment protocols toward dynamic, precision medicine. Realizing this vision necessitates interdisciplinary collaboration to engineer robust, generalizable, and interpretable AI systems. Key priorities include the establishment of large-scale multimodal data repositories, the advancement of explainable AI (XAI) frameworks, the execution of rigorous prospective clinical trials to validate efficacy, and the implementation of adaptive regulatory and ethical guidelines. By systematically addressing these barriers, AI can evolve from a mere analytical tool into an indispensable clinical partner, ultimately optimizing patient outcomes.

## 1. Introduction

Stroke remains a leading cause of mortality and permanent disability globally [1,2,3]. Among stroke subtypes, spontaneous intracerebral hemorrhage (ICH)—characterized by the acute rupture of a cerebral vessel and subsequent bleeding into the surrounding parenchyma—constitutes 10–15% of all strokes worldwide. Despite its lower incidence compared to ischemic stroke, ICH is disproportionately responsible for a substantial burden of stroke-related mortality and long-term morbidity [4,5,6]. Notwithstanding advancements in acute neurocritical care, the prognosis for patients with ICH remains dismal: one-month mortality rates approach 40%, and fewer than 20% of survivors achieve functional independence at six months [7,8]. This unfavorable clinical trajectory is driven by a complex pathophysiological cascade, primarily involving acute hematoma expansion, perihematomal edema (PHE) formation, and secondary neuroinflammatory injury [8,9].

Critical decision-making in ICH management—encompassing hematoma evacuation, hemodynamic control, prognostication, and rehabilitation planning—is heavily contingent upon the rapid and precise interpretation of neuroimaging findings and clinical markers. However, current clinical paradigms face inherent limitations. The manual volumetric segmentation of hematomas and PHE on computed tomography (CT) scans is labor-intensive, susceptible to inter-rater variability, and frequently unfeasible in time-sensitive emergency settings [10,11]. Furthermore, the prediction of clinical outcomes and secondary complications, such as hematoma expansion or stroke-associated pneumonia, traditionally relies on clinical grading scales that often lack the granularity required for individual-level precision [12,13].

In this context, AI, particularly machine learning (ML) and deep learning (DL), has emerged as a disruptive technology capable of addressing these long-standing clinical bottlenecks. Recent years have witnessed an explosive growth in AI applications for ICH, transitioning from basic task automation to generating data-driven insights for complex clinical dilemmas. AI algorithms now demonstrate expert-level proficiency in the automated segmentation of intraparenchymal and intraventricular hemorrhages, as well as PHE, facilitating rapid, quantitative, and reproducible volumetric analyses [14,15]. Beyond image processing, ML models are increasingly deployed to predict functional outcomes, in-hospital mortality, and specific sequelae with high fidelity by integrating multimodal datasets encompassing imaging, electronic health records, and genomic profiles [16]. Pioneering research is also exploring AI-augmented surgical planning, leveraging augmented reality (AR) navigation systems and optimizing stereotactic catheter puncture trajectories [11,17].

However, the integration of these powerful computational tools into routine clinical workflows is non-trivial. Challenges regarding model generalizability, data privacy, “black-box” opacity, and ethical accountability must be rigorously addressed before AI can be seamlessly translated to the bedside [18,19,20,21].

Therefore, this comprehensive review aims to synthesize the rapid advancements in AI-driven ICH management. We delineate the key opportunities AI presents across the clinical continuum—from initial neuroimaging interpretation to long-term outcome prediction. Subsequently, we critically examine the significant systemic and technical barriers that hinder widespread clinical adoption. By providing a balanced perspective on both the potential and the pitfalls of these technologies, this review seeks to inform clinicians and researchers, ultimately guiding the future development of robust, equitable, and clinically impactful AI solutions for ICH management.

## 2. Materials and Methods

This article is designed as a narrative review aimed at providing a conceptually oriented synthesis of recent developments in artificial intelligence (AI) applications for intracerebral hemorrhage (ICH).

To ensure broad coverage of relevant literature, a structured search of major biomedical and engineering databases, including PubMed, Web of Science, and IEEE Xplore, was conducted. The search focused on studies addressing AI-related methodologies and technologies relevant to ICH, such as machine learning, deep learning, neuroimaging analysis, prognostic modeling, surgical navigation, and brain–computer interfaces.

Articles were considered based on their relevance, methodological contribution, and conceptual significance to the evolving landscape of AI in ICH research and clinical practice. Emphasis was placed on representative studies that illustrate key technological directions, methodological challenges, and translational considerations.

Given the narrative nature of this review and the heterogeneity of the included literature, no formal systematic review framework, eligibility criteria, or risk-of-bias assessment was applied. Instead, the review prioritizes thematic integration, interpretative analysis, and critical discussion of emerging trends, limitations, and future perspectives.

## 3. AI-Powered Clinical and Translational Applications in ICH

### 3.1. Automated Quantification and Diagnostic Biomarker Discovery

Computed tomography (CT) remains the gold standard for the acute diagnostic evaluation of ICH, facilitating the rapid identification of acute hemorrhage in emergency settings [22]. Crucially, hematoma volume, morphological characteristics, and the extent of perihematomal edema (PHE) serve as primary determinants of patient prognosis [23,24,25]. Conventional techniques for quantifying these parameters—such as the manual ABC/2 volumetric estimation or slice-by-slice region-of-interest manual segmentation—are constrained by significant methodological limitations [26]. These approaches are labor-intensive, exhibit diminished accuracy when applied to irregularly shaped hematomas, and are subject to substantial inter-rater variability, thereby limiting their utility in time-sensitive acute care environments.

To circumvent these bottlenecks, deep learning (DL) architectures, notably U-Net and its derivatives, have been engineered for the automated and high-fidelity delineation of ICH compartments [15,16,27]. For instance, Kuang et al. introduced an uncertainty-aware DL framework employing cross-task supervision for PHE segmentation. By leveraging slice-level labels and pixel-wise structural ICH annotations to generate high-fidelity pseudo-labels, this model achieved Dice similarity coefficients commensurate with fully supervised algorithms [14]. Similarly, Zhang et al. developed a contrastive learning-based architecture featuring dynamic memory banks for the segmentation of minute “spot signs” on multiphase CT angiography (CTA). This model achieved a mean Dice score of 0.638, underscoring the viability of automated volumetric analysis in forecasting hematoma expansion [28]. Expanding beyond hematoma and spot sign analysis, Ma et al. curated the first public benchmark CT dataset (PHE-SICH-CT-IDS) dedicated to PHE segmentation, detection, and radiomic feature extraction. This repository facilitates the standardized benchmarking of DL models, thereby catalyzing subsequent methodological advancements [29].

The quantitative outputs derived from these segmentation networks function as pivotal inputs for downstream diagnostic and prognostic pipelines, enabling robust, data-driven clinical decision support [15,16]. Crucially, the predictive utility of AI extends beyond static anatomical mapping to the identification of dynamic imaging biomarkers. For example, Chen et al. deployed a 3D U-Net to co-segment the hematoma and PHE, formalizing the concept of “Delayed Perihematomal Edema Expansion” (DPE). They identified an absolute PHE expansion exceeding 3.34 mL between days 4–7 and 8–14 post-ictus as a robust predictor of poor functional outcomes [16]. Such model-derived biomarkers equip clinicians with objective criteria to stratify high-risk cohorts who may require targeted therapeutic interventions [14,16]. Concurrently, the rigorous quantification of PHE via uncertainty-aware DL networks introduces a critical dimension to surgical planning by estimating the trajectory of secondary injury and its impending impact on adjacent eloquent cortex [14]. Furthermore, Tong et al. illustrated the direct translation of segmentation accuracy into therapeutic action: their 3D U-Net model not only yielded high Dice scores for intraparenchymal and intraventricular hemorrhages but also facilitated the optimization of stereotactic catheter trajectories, achieving a 96% suitability rate (defined by a centroid deviation < 10 mm or Dice > 0.8) [15]. Together, these innovations delineate a closed-loop, AI-augmented workflow wherein automated segmentation seamlessly informs both prognostic stratification and neurosurgical intervention [15,28].

The convergence of AI-driven analytics with advanced neuroimaging is forging a transformative ecosystem in ICH management, concurrently elevating prognostic precision and democratizing clinical access. Automated segmentation algorithms yield indispensable quantitative biomarkers—ranging from spot sign volumes for predicting hematoma expansion to PHE expansion rates correlated with functional deficits [15,16,28]. These imaging-derived indices are increasingly fused with clinical metadata to engineer sophisticated prognostic models, such as those forecasting 90-day functional recovery or the risk of stroke-associated pneumonia, thereby expediting the triage of high-risk patients [30,31]. This analytical capacity is further augmented by the advent of portable, low-field magnetic resonance imaging (MRI) systems. Empowered by DL-based image reconstruction, these devices demonstrate robust diagnostic accuracy, rendering advanced neuroimaging viable in resource-constrained environments or at the bedside of hemodynamically unstable patients [32]. The synergistic interplay among specialized benchmark datasets, uncertainty-aware segmentation algorithms, and portable imaging modalities establishes a cohesive pipeline spanning from raw image acquisition to actionable clinical intelligence [14,29]. Underpinned by explainable AI (XAI) frameworks and cross-modal architectures, this integrated paradigm ensures that data-driven insights can be reliably deployed across diverse clinical settings, ultimately propelling equitable and personalized care for patients with ICH [33,34].

Beyond conventional data augmentation techniques, generative adversarial networks (GANs) have recently emerged as a powerful paradigm for synthesizing high-fidelity medical images to address data scarcity and class imbalance. In the neuro-oncology domain, Pix2Pix GAN-based frameworks have been shown to generate realistic contrast-enhanced MRI sequences, significantly improving brain tumour classification performance [35]. Similarly, conditional deep convolutional GANs (cDCGANs) have been successfully employed to produce synthetic brain tumour datasets that, when combined with real images, enhance the robustness and generalizability of DL classifiers [36]. These advances hold considerable promise for ICH imaging, where annotated datasets are often limited and hemorrhage or edema regions are highly imbalanced. Adapting such generative augmentation strategies to non-contrast CT or portable MRI could further improve segmentation accuracy and prognostic model stability, particularly in low-resource settings.

### 3.2. Intelligent Surgical Navigation and Brain–Computer Interface

The integration of AI into the surgical management of ICH is refining both decision-making and technical execution. By building upon automated segmentation, AI provides a dynamic foundation for real-time surgical navigation. Advanced visualization systems, particularly augmented reality (AR) platforms, create detailed 3D virtual models of the hemorrhage and critical brain structures, which are overlaid directly onto the surgeon’s field of view [17]. This integration offers sub-millimeter guidance for tasks ranging from macroscopic trajectory planning to microscopic endoscopic evacuation, effectively bridging preoperative planning with intraoperative action. This capability allows surgeons to navigate complex anatomy with enhanced precision, thereby potentially minimizing damage to eloquent brain regions. Furthermore, the synergy of AI with portable imaging technologies is broadening access to advanced surgical planning. Portable MRI systems, enhanced by DL reconstruction algorithms, have demonstrated high sensitivity and specificity for ICH detection at the bedside in early studies [32]. This innovation could make sophisticated neuroimaging more feasible in resource-limited settings or for critically ill patients who cannot be transported, suggesting a pathway toward more integrated care from point-of-care image acquisition to AI-driven analysis.

Brain–computer interface (BCI) technologies represent one of the most intriguing frontiers in neurorehabilitation; however, their application in intracerebral hemorrhage remains largely experimental. Unlike imaging-based AI tools that are increasingly approaching clinical integration, BCI systems are primarily supported by proof-of-concept studies, small-scale clinical investigations, and translational research. Therefore, the following discussion of BCIs should be interpreted within the context of emerging rather than established clinical practice.

The conceptual foundation for BCI in ICH care lies in addressing the limitations of conventional rehabilitation. While surgical evacuation addresses the macroscopic anatomical problem, a patient’s ultimate quality of life depends on neural functional remodeling—a process where BCI technology may eventually offer novel therapeutic avenues [37]. By establishing a direct communication pathway between the brain and external devices that bypasses peripheral nerves and muscles, BCI could provide a platform for real-time insight into brain states, quantification of neural function, and intervention in neural activity [38].

At the level of neurological function assessment, preliminary research suggests that multimodal BCIs integrating electroencephalography (EEG) and functional near-infrared spectroscopy (fNIRS) may be capable of non-invasively capturing aspects of brain function. For instance, a novel hybrid EEG-fNIRS BCI framework incorporating Wasserstein metric-based transfer learning has been developed, with early results suggesting it may help quantify differences in neurosignals between healthy individuals and ICH patients, potentially enhancing cross-subject decoding performance in small-scale studies [39]. Such approaches are being explored as possible tools for addressing individual variability and objectively assessing neurological function changes before and after surgery, though these applications remain investigational.

In the domain of motor function restoration, preliminary studies suggest that BCI could offer novel approaches for patients with severe paralysis who may not benefit adequately from conventional rehabilitation. Minimally invasive endovascular BCIs, such as the Stentrode device (Synchron, New York, NY, USA), represent a promising technological alternative to traditional implanted systems, with early feasibility studies demonstrating stable signal recording and potential for functional restoration in severely paralyzed patients in home environments [40]. However, these findings derive from highly selected patient populations and require confirmation in larger, more diverse cohorts. Similarly, BCI-driven neuromuscular electrical stimulation (NMES) has shown initial promise for chronic paresis after stroke in proof-of-concept investigations. The approach—utilizing the brain’s own motor intent to drive electrical stimulation—may theoretically promote functional reorganization of motor pathways, but robust evidence from adequately powered trials is currently lacking [41].

Looking forward, precision surgical techniques centered on AI and AR, increasingly accessible diagnostic tools represented by portable imaging, and emerging neurorehabilitation technologies grounded in BCI research collectively hint at an evolving therapeutic landscape for ICH [42]. If validated in future research, these approaches could one day contribute to a more comprehensive care continuum, though at present they remain largely within the domain of clinical investigation rather than routine practice [43].

### 3.3. Proactive Prognostication

In neurocritical care, the cornerstone of proactive prognostication is the utilization of multimodal clinical data to prospectively identify patients at an elevated risk of adverse trajectories, thereby facilitating targeted and preemptive interventions. The operationalization of this paradigm relies implicitly on the development and rigorous validation of robust predictive algorithms. Recently, ML and allied AI technologies have emerged as primary engines for constructing high-fidelity prognostic models. Their efficacy stems from an intrinsic capacity to process and map high-dimensional, non-linear biomedical data [44,45].

Throughout the acute phase of ICH, patients are vulnerable to a spectrum of severe complications—including nosocomial infections, deep vein thrombosis, and acute symptomatic seizures—all of which independently exacerbate morbidity and mortality. Regarding hospital-acquired infections, stroke-induced immunosuppression and subsequent gut microbiota dysbiosis render ICH patients uniquely susceptible to systemic pathogens [46,47]. Furthermore, the onset of severe infections, such as sepsis, initiates a deleterious feedback loop with the primary central nervous system injury. This exacerbates the systemic inflammatory response syndrome (SIRS) and precipitates multiple organ dysfunction, significantly amplifying mortality and long-term disability [48,49]. Consequently, the preemptive and accurate stratification of complication risk is an indispensable component of proactive neurocritical management.

Conventional risk stratification tools typically depend on isolated biomarkers or reductionist clinical scoring systems, which frequently suffer from suboptimal sensitivity and specificity. In contrast, ML architectures can seamlessly ingest vast, heterogeneous data matrices available at admission—encompassing demographics, continuous vital signs, comprehensive laboratory panels, comorbidities, and acute interventions—to autonomously identify latent prognostic patterns and generate early clinical alerts. Extensive literature confirms that ensemble learning algorithms (e.g., Random Forests, Gradient Boosting Machines) and Recurrent Neural Networks (RNNs) consistently outperform traditional logistic regression models in forecasting diverse clinical end-points [50,51].

A compelling demonstration of this is provided by Tang et al. [52], who systematically engineered and benchmarked multiple ML algorithms to predict sepsis risk among ICH patients utilizing expansive public intensive care unit (ICU) registries. Their analysis revealed the superiority of the Random Forest model, which maintained excellent discriminatory capacity and generalizability across independent internal and external validation cohorts. This performance aligns with the architectural strengths of Random Forests, specifically their resilience to missing data, their ability to model complex multimodal interactions, and their inherent robustness against overfitting in noisy biomedical datasets [53], establishing them as an optimal substrate for prognostic modeling.

Beyond binary risk stratification, ensuring model interpretability is paramount for translating predictions into actionable clinical interventions. The integration of post hoc explanatory frameworks, such as SHapley Additive exPlanations (SHAP), in the aforementioned study elucidated the primary drivers of sepsis risk, highlighting variables such as fluid and electrolyte imbalances, leukocytosis, supplemental oxygen requirements, depressed Glasgow Coma Scale (GCS) scores, and acute kidney injury. These algorithmically derived insights are corroborated by established pathophysiological literature: severe electrolyte derangements independently predict ICU mortality [54], concurrent acute pneumonia exponentially increases the risk of downstream post-stroke sequelae [55], and invasive monitoring or respiratory support are well-established vectors for nosocomial pathogens [56]. Thus, advanced ML models serve a dual imperative: they provide highly sensitive early warning systems while simultaneously isolating critical pathophysiological drivers, thereby directing clinicians toward precision, mechanism-targeted interventions.

Beyond automated image analysis, a critical clinical priority is the accurate prognostication of patient survival and long-term functional independence. Traditional risk stratification tools, such as the classic ICH Score, are clinically pragmatic but often lack the precision required for individualized long-term functional prediction. Recently, machine learning models have demonstrated superior discriminatory ability in forecasting both short-term mortality and long-term functional outcomes.

For instance, studies utilizing ensemble learning algorithms (e.g., Random Forest, XGBoost, and CatBoost) integrated with multimodal data—including admission clinical metrics, laboratory values, and radiomic features—have consistently outperformed traditional scores in predicting 90-day modified Rankin Scale (mRS) scores and mortality rates [57]. Research by Guo et al. highlighted that ML models could achieve an Area Under the Curve (AUC) of nearly 0.89 for predicting 90-day functional recovery, providing a significantly more robust prognostic profile than the conventional ICH Score. Furthermore, predictive models have been specifically tailored for vulnerable populations, such as elderly patients, where predicting the 6-month Glasgow Outcome Scale (GOS) is arguably more critical than mortality alone for establishing realistic goals of care [58]. Similarly, the integration of real-world Electronic Health Record (EHR) data with raw CT imaging has enabled models to achieve superior discrimination and calibration in predicting in-hospital mortality compared to standard clinical grading scales [59]. By uncovering complex, non-linear synergies among clinical variables that traditional regression models might miss, AI equips clinicians with dynamic prognostic tools that can continuously update as the patient’s condition evolves, thereby facilitating more personalized and transparent family counseling and therapeutic decision-making.

### 3.4. Decoding Disease Mechanisms in Fundamental Research

AI is catalyzing a paradigm shift in fundamental ICH research by empowering a multi-scale, data-driven approach to deciphering underlying pathophysiology. At the molecular echelon, AI-driven structural biology platforms, notably AlphaFold (Google DeepMind, London, UK) and RoseTTAFold (University of Washington, Seattle, WA, USA), generate unprecedented, atomic-resolution models of proteins implicated in ICH pathogenesis. In the context of cerebral amyloid angiopathy (CAA)—a predominant etiology of lobar ICH—these computational tools accelerate the rational design of novel therapeutics aimed at enhancing amyloid-β clearance and restoring cerebrovascular integrity [60,61]. Extending beyond static structural conformations, utilizing AI to model dynamic protein–protein interaction (PPI) networks is elucidating critical molecular cascades, such as the complement system activation that propagates secondary neuroinflammation following hemorrhage [62].

Within systems biology, ML architectures are increasingly deployed to interrogate high-dimensional “multi-omics” datasets to extract latent biological signatures. A paramount application involves the discovery of robust diagnostic and prognostic biomarkers from peripheral biofluids. Specifically regarding CAA, circulating microRNAs (miRNAs) have emerged as highly promising non-invasive biomarkers and potential therapeutic targets, mechanistically bridging the gap between progressive vascular amyloid deposition and acute hemorrhagic rupture [63]. Building upon this framework, ML algorithms have successfully identified distinct circulating miRNA signatures in patient serum that correlate strongly with the risk of hematoma expansion and long-term functional recovery profiles [64]. Furthermore, the fusion of multi-omics data (e.g., transcriptomics, proteomics) via deep learning models is unmasking novel pathogenic networks. For example, algorithmic identification of correlations between iron-metabolism gene expression and delayed PHE underscores ferroptosis as a highly viable therapeutic target for mitigating secondary brain injury [65].

A particularly potent methodological advancement is the integration of bulk and single-cell RNA sequencing (scRNA-seq) datasets utilizing specialized ML pipelines. This synergy not only isolates critical differentially expressed genes (DEGs)—such as *ANXA2* and *COTL1*, which are upregulated post-ictus and mechanistically linked to microglial activation and oxidative stress—but also facilitates the deconvolution of immense cellular heterogeneity, mapping these molecular signatures precisely to their cellular origins (e.g., microglia versus reactive astrocytes) [66]. Advanced computational techniques, such as pseudotime trajectory inference algorithms applied to scRNA-seq matrices, map the dynamic temporal transition of microglia from a homeostatic resting state to a pro-inflammatory phenotype. This high-resolution temporal modeling meticulously characterizes the transcriptional drivers governing this pathogenic phenotypic switch [67]. Ultimately, this AI-powered, multi-scale analytical framework provides an indispensable mechanism for deconvoluting the intricate cellular kinetics and molecular pathways driving secondary neural injury, thereby laying the groundwork for the next generation of precision, cell-type-specific neurotherapeutics.

**Figure 1:**
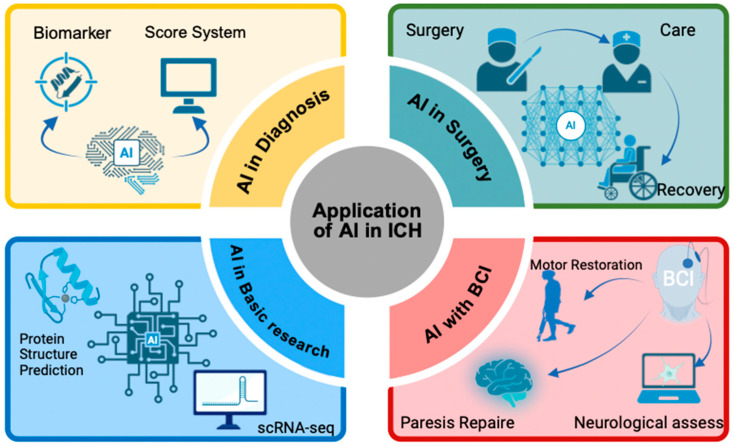
The applications of AI in ICH.

Figure 1 illustrates from four perspectives: diagnosis, treatment, brain–computer interfaces, and basic research.

### 3.5. Critical Appraisal of Current Evidence

The technological advances surveyed across Section 3.1, Section 3.2, Section 3.3 and Section 3.4 illustrate the remarkable breadth of AI applications in ICH research. However, a critical examination reveals substantial heterogeneity in methodological rigor and clinical readiness.

In automated image segmentation, while deep learning models frequently report Dice coefficients exceeding 0.9, most studies remain retrospective, single-center designs utilizing curated datasets that may not reflect real-world heterogeneity. Sample sizes vary considerably, with some studies training on fewer than 200 scans. External validation—the gold standard for assessing transportability—remains exceptional rather than routine. Crucially, few studies demonstrate that improved segmentation accuracy translates into meaningful changes in clinical decision-making or patient outcomes.

For prognostic prediction models, although machine learning approaches often outperform traditional regression in internal validation, performance degradation upon external validation is well-documented. Most models are developed on modest-sized datasets and lack temporal or geographic validation. Calibration—essential for clinical utility—is frequently underreported, and prospective studies demonstrating improved patient management remain absent.

Surgical navigation research is dominated by technical feasibility studies with sample sizes rarely exceeding 30 patients, precluding robust conclusions about safety or efficacy. BCI research in ICH remains at the proof-of-concept stage, involving highly selected patients under controlled conditions without adequate control groups.

Collectively, the field is characterized by exploratory research with limited confirmatory evidence. The path from technical proof-of-concept to clinically validated tool requires rigorous external validation, prospective evaluation against relevant comparators, and demonstration of meaningful impact on patient-centered outcomes.

## 4. Navigating the Translational Pathway: Multifaceted Challenges in Deploying AI for ICH

### 4.1. Data Foundations: Heterogeneity, Scarcity, and Privacy

The clinical realization of AI in ICH management is intrinsically contingent upon the quality, scale, and diversity of the underlying training data. The translational journey from algorithm to bedside, however, is impeded by formidable data-related challenges that fall into two primary domains: intrinsic data quality and systemic data governance.

The first domain pertains to the intrinsic limitations of the data itself. A paramount hurdle is the profound heterogeneity and variable quality inherent to real-world medical data. Although AI models frequently achieve high accuracy in controlled, in silico research settings, their real-world performance is heavily reliant on standardized imaging protocols and meticulous ground-truth annotations [27]. However, the limited scale of existing annotated datasets remains a fundamental bottleneck. Many models are currently trained on small, single-center cohorts that fail to capture the vast pathological spectrum and demographic diversity of ICH patients, fundamentally capping their potential for broad clinical application. Routine clinical practice is characterized by pronounced inter-institutional variability in scanner hardware and acquisition parameters. This discrepancy invariably engenders a substantial “generalization gap” (or domain shift) when models are exposed to out-of-distribution data. This challenge is exacerbated by the intricacies of harmonizing complex, multimodal data streams—a systemic limitation analogously observed in the deployment of multimodal large language models [68]. Crucially, achieving a comprehensive algorithmic understanding of ICH—a pathophysiologically multifaceted disease—necessitates the seamless integration of multimodal inputs, encompassing perfusion neuroimaging, laboratory biomarkers, genomic profiles, and continuous physiological telemetry [69].

This heterogeneity paradigm extends to BCI applications, where the decoding of motor intention is contingent upon stable neural signals that are frequently and idiosyncratically disrupted by the underlying ICH pathophysiology [43]. Beyond data variability, the curation of ground-truth annotations constitutes a significant bottleneck. The manual delineation of complex, ambiguous structures such as PHE is highly resource-intensive and inherently susceptible to inter-rater variability. This introduces label “noise” that can severely misdirect model optimization [70]. Similarly, the labeling of neural states for BCI training paradigms is a laborious, subjective process that impedes the development of robust neural decoders [71]. Methodological initiatives, such as the CLAIM checklist, aim to mitigate these deficiencies by mandating standardized reporting of data provenance and labeling protocols, thereby augmenting transparency and reproducibility [72].

The second domain encompasses the systemic tension between collaborative data pooling and patient privacy. Multi-institutional collaborations are indispensable for curating datasets with sufficient diversity to train robust, generalizable models, as evidenced by the successful external validation of clinical tools like the ICH-LR2S2 score [31]. However, the aggregation of sensitive neuroimaging and genomic data is strictly governed by ethical mandates and regulatory frameworks, such as HIPAA. This privacy barrier is profoundly acute in BCI research, which relies on exceptionally sensitive electrocorticographic or intracortical telemetry [73]. Although the establishment of public benchmark repositories, such as the PHE-SICH-CT-IDS dataset, represents a commendable stride toward standardization, such initiatives alone cannot circumvent the overarching privacy barrier. Collectively, these factors precipitate a severe fragmentation of the medical data ecosystem. Clinical data siloed within Electronic Health Records (EHRs), Laboratory Information Systems (LIS), and Picture Archiving and Communication Systems (PACS) are not only structurally disparate but also plagued by profound semantic heterogeneity and limited interoperability [74].

Furthermore, a widespread lack of reproducibility exacerbates this fragmentation. Many studies rely on closed-source code, obscure hyperparameter settings, and poorly documented preprocessing pipelines, preventing independent researchers from validating or building upon their findings. Establishing an ecosystem of open-source algorithms and adherence to rigorous reporting standards, such as the TRIPOD-AI and STARD-AI guidelines, is imperative to ensure methodological transparency and reproducible science. Consequently, this opacity creates a formidable obstacle to multi-institutional research and large-scale data aggregation.

Generative models as a pathway to data enrichment. One promising avenue to overcome data scarcity and label imbalance is the use of generative adversarial networks (GANs) to create synthetic but anatomically plausible imaging data. Recent studies in brain tumour classification have demonstrated that Pix2Pix GAN-based augmentation can generate high-quality MRI slices that preserve pathological features, leading to substantial gains in classifier sensitivity and specificity [35]. Likewise, conditional deep convolutional GANs (cDCGANs) have been employed to produce large-scale synthetic tumour datasets that, when used for pre-training or joint training, mitigate overfitting and improve cross-dataset generalization [36]. Although GAN-based augmentation has not yet been systematically evaluated in ICH imaging, its successful application in other hemorrhagic or edematous lesion contexts suggests strong translational potential. Integrating such generative techniques with federated learning (FL) [75] or uncertainty-aware segmentation frameworks could simultaneously address data heterogeneity, privacy constraints, and class imbalance—representing a high-priority direction for future ICH-AI research.

### 4.2. Algorithmic Barriers: The Imperatives of Interpretability and Resilience

Even assuming the availability of robust datasets, the clinical translation of AI is impeded by formidable algorithmic barriers, predominantly the ubiquitous “black-box” problem. The highly complex, non-linear feature mappings inherent to DL architectures frequently yield predictive outputs that are inscrutable to clinicians. This opacity fosters deep-seated distrust and precludes the integration of AI into high-stakes, acute clinical workflows [76,77]. This deficit of trust extends beyond the medical team to patients and their surrogates, potentially eroding confidence in both the algorithmic tool and the attending physician. Consequently, advancements in Explainable AI (XAI) are imperative. As highlighted in foundational literature, the inability to elucidate model rationale is a primary barrier to adoption in safety-critical domains like healthcare, necessitating methodologies that render AI reasoning transparent, interpretable, and clinically contestable [78].

Within the BCI domain, this opacity constitutes a critical impediment; clinicians are understandably hesitant to rely on systems that translate neural activity into rehabilitative actuation without providing a biologically plausible rationale [79,80]. As Reyes et al. articulate, bridging the chasm between purely technical feature attributions and clinically actionable insights remains a paramount challenge [81]. Accordingly, the computational research community is increasingly pivoting toward robust XAI frameworks. For instance, the H-SYSTEM architecture enhances transparency by anchoring its inferences within a structured medical knowledge graph, enabling clinicians to audit its deductive logic [33]. Similarly, the utilization of SHapley Additive exPlanations (SHAP) to quantify the prognostic weight of specific inputs—such as the NIHSS score or initial hematoma volume—facilitates the transformation of an opaque prediction into a transparent, individualized clinical risk profile [82]. In BCI development, emerging XAI techniques are deployed to visualize precisely which neural features (e.g., specific oscillatory bands or spatial topographies) drive the decoded kinematic output, thereby bolstering clinician confidence in the neural interface [83]. This paradigm shift—from isolated prediction to comprehensible clinical justification—is an absolute prerequisite for establishing a reliable human-AI collaborative partnership.

Parallel to the imperative of interpretability is the critical requirement for algorithmic resilience. An AI model must maintain performance stability not only across disparate healthcare networks but also longitudinally, dynamically adapting to temporal shifts in clinical protocols and population demographics [84]. BCI systems encounter a unique dimension of this challenge: they must adapt not only to inter-institutional variances but also to the dynamic, continuous neuroplastic alterations occurring within an individual patient’s cortex throughout the rehabilitation trajectory [85]. The CONSORT-AI extension emphasizes this necessity by mandating rigorous external validation and the meticulous reporting of the specific clinical deployment environment in AI-focused clinical trials [86].

A fundamental architectural limitation undermining the resilience of contemporary models is their heavy reliance on statistical associations, which frequently leads to the assimilation of spurious correlations derived from biased training manifolds. Causal representation learning—which mathematically decouples stable, causal mechanisms from unstable, non-causal artifacts—offers a robust trajectory toward models that generalize reliably across diverse hospital networks [87,88]. Ascending Judea Pearl’s hierarchy of causal reasoning—progressing from mere association to intervention, and ultimately to counterfactual inference—is vital for resolving complex clinical questions regarding treatment efficacy. However, achieving this represents a monumental methodological leap [89]. This evolution necessitates capabilities such as Continual Learning, enabling models to assimilate new data and update parameters sequentially as patient pathophysiology evolves—a functional requirement that is far from standard practice [90]. Encouragingly, novel paradigms such as self-supervised learning are emerging to address these deficits by pre-training on massive, unlabeled datasets to extract fundamental, invariant representations of neuroanatomy and underlying pathology [91]. Concurrently, adaptive BCI architectures that autonomously recalibrate their decoding algorithms in response to the user’s fluctuating neural signatures are under active development to ensure long-term kinematic fidelity [92]. Furthermore, while the fusion of Reinforcement Learning (RL) with causal frameworks holds immense promise for optimizing personalized, dynamic treatment regimens, guaranteeing the safety and stability of RL agents in unpredictable clinical environments remains a formidable challenge [93]. Ultimately, the objective is to transcend models optimized solely for in silico test-set accuracy, pivoting toward the engineering of resilient, transparent, and highly reliable clinical partners.

### 4.3. Clinical Integration: Augmenting Workflows

A technically superior AI architecture is rendered clinically inert if it remains isolated from established medical workflows. The ultimate metric of utility lies in the system’s seamless integration into the high-velocity, high-stakes environment of neurocritical ICH management. As emphasized by Park and Han, demonstrating in silico diagnostic accuracy is merely the preliminary step; the definitive measure of success is the technology’s tangible impact on workflow efficiency and downstream patient outcomes [94]. This integration poses a significant hurdle for BCI-based neurorehabilitation platforms. These systems frequently demand substantial setup times, highly specialized hardware, and dedicated, intensive oversight by physical therapists, complicating their adoption into fast-paced inpatient units or home-care settings [95]. Similarly, sophisticated platforms such as AR-augmented neuroendoscopic navigators provide unprecedented anatomical visualization but frequently necessitate disruptive alterations to deeply entrenched surgical protocols and demand prohibitive capital investments [17]. Realizing seamless integration mandates a rigorously clinician-centric design philosophy and sustained, deep interdisciplinary collaboration—dynamics that are notoriously difficult to orchestrate at scale [90].

Conversely, the most successfully adopted AI tools are those designed to implicitly augment, rather than explicitly interrupt, existing workflows [96]. Automated segmentation algorithms that render precise volumetric quantifications of hematomas within minutes of CT acquisition, or point-of-care web-based prognostic calculators, exemplify this principle. They optimize efficiency and support, rather than supplant, clinical judgment [15,27,31]. Correspondingly, the most viable BCIs for clinical translation are envisioned as assistive adjuncts that augment the neuro-therapist’s capabilities—for instance, by delivering objective, real-time physiological metrics of patient engagement and motor intent during sessions—rather than attempting to autonomously replace human-guided rehabilitation [43].

This principle of intelligent augmentation defines the evolving paradigm of the human-AI partnership in medicine. The future trajectory of AI in ICH is not characterized by autonomous agents replacing neurocritical care teams, but rather by synergistic “collaborative intelligence” [97]. Within this framework, the AI functions as a high-throughput computational assistant, executing data-intensive tasks such as high-dimensional pattern recognition and rapid quantitative volumetrics. This offloading liberates the clinician to concentrate on higher-order cognitive functions: synthesizing complex clinical gestalts, applying nuanced experiential intuition, navigating complex ethical dilemmas, and managing the inherently empathetic human dimension of patient care. In a BCI-mediated rehabilitation context, this translates to the neural interface reliably decoding the patient’s motor intent to actuate robotic orthotics, while the human therapist focuses on dynamically tailoring the overarching rehabilitation strategy, providing psychological motivation, and ensuring optimal biomechanical kinematics [98]. The successful deployment of centralized, AI-driven triage algorithms within distributive stroke networks—which optimize critical resource allocation by intelligently routing acute patients—perfectly epitomizes this synergistic potential [99]. For AI to achieve deep, sustained embedding within routine clinical practice, it must validate itself not as an infallible, standalone diagnostician, but as an integrated, indispensable component of a cohesive multidisciplinary care team.

### 4.4. Regulatory Lag and Evidence Gap

The exponential pace of AI innovation has drastically outstripped the maturation of the legal and regulatory frameworks required to govern its safe, equitable, and efficacious deployment. This profound regulatory lag constitutes a critical translational bottleneck [100]. As articulated by Topol, the dynamic, continuously evolving nature of modern DL models fundamentally contradicts the rigid, traditional regulatory pathways historically designed for the approval of static medical hardware [97]. BCI technologies are situated at the absolute frontier of this regulatory challenge. They are frequently classified as invasive medical devices, yet they inherently possess the adaptive, iterative capabilities characteristic of Software-as-a-Medical-Device (SaMD), thereby engendering significant ambiguity within existing approval paradigms. As AI systems acquire increasing clinical autonomy—evidenced in domains such as AI-driven de novo drug discovery and autonomous robotic surgery—the mandate for auditable, highly interpretable, and mechanically controllable architectures becomes non-negotiable, thereby further convoluting the regulatory landscape [101].

The advent of these highly complex, autonomous systems underscores the urgent necessity for fundamentally novel regulatory architectures. Leading authorities advocate for the implementation of adaptive regulatory frameworks and rigorous, continuous post-market surveillance mechanisms specifically tailored for continuously learning AI. These are essential to guarantee longitudinal safety and algorithmic efficacy throughout the software’s lifecycle [102]. Concurrently, a glaring evidence gap persists. There remains a stark paucity of prospective, multi-center randomized controlled trials (RCTs) capable of conclusively demonstrating that AI-driven interventions statistically improve hard clinical end-points (e.g., mortality, functional independence) for patients with ICH. The evidence base underpinning clinical BCI applications is similarly nascent; there is an exigent need for adequately powered, large-scale trials that move beyond validating mere technical efficacy (e.g., decoding accuracy) to definitively proving substantial improvements in activities of daily living (ADLs) and long-term quality of life [103]. This methodological gap mirrors broader translational challenges within cerebrovascular medicine, prompting the proposition of adaptive, pragmatic trial platforms designed to systematically and rapidly evaluate novel interventions [104]. The implementation of the CONSORT-AI guidelines represents a foundational step forward, establishing a stringent framework for the transparent, reproducible reporting of AI interventions. This rigor is an absolute prerequisite for generating the high-fidelity clinical evidence demanded by regulatory bodies [86].

### 4.5. Ethical Governance: Accountability and Bias

Beyond clinical and technical validation, the establishment of a comprehensive governance infrastructure is critical to navigate the profound ethical, legal, and sociotechnical dilemmas intrinsic to medical AI. The deployment of autonomous algorithms for highly sensitive clinical tasks—such as mortality prognostication or genomic risk stratification—precipitates complex questions of medical liability and accountability: when a deep learning system generates an erroneous, deleterious recommendation, where does the ultimate legal culpability reside [102]? Establishing unequivocal legal accountability frameworks is therefore paramount, particularly within the high-acuity domain of ICH management. A sophisticated, distributed responsibility matrix must meticulously delineate liability among institutional data providers, algorithmic developers, the attending clinicians, and, theoretically, the autonomous AI agents themselves. Proactively addressing medical malpractice liability for algorithmically induced diagnostic errors, mitigating systemic algorithmic bias against underrepresented demographic cohorts, and preventing the insidious erosion of patient autonomy represent urgent ethical imperatives [101].

In the context of BCIs, which establish a direct conduit to the central nervous system, these ethical quandaries are exponentially magnified. They encompass profound philosophical concerns regarding human agency and identity, alongside acute privacy risks concerning the potential for high-resolution neurodata to inadvertently expose highly sensitive cognitive or emotional states [105,106]. Furthermore, algorithms trained on historically biased or demographically skewed datasets can inadvertently perpetuate, or mathematically amplify, deeply entrenched healthcare disparities, elevating algorithmic fairness and sociodemographic equity to central concerns [107]. Inherent data biases—encompassing selection bias, information bias, and unmeasured confounding—profoundly degrade model generalizability, frequently resulting in clinically unacceptable, differential performance across distinct racial, socioeconomic, or gender stratifications [107,108]. Future translational research must rigorously integrate robust bias detection and mitigation pipelines. This includes the application of causal inference methodologies and the intentional curation of highly diverse, globally representative training cohorts. The adoption of causal fairness frameworks is instrumental in mathematically guaranteeing that AI systems do not exacerbate existing disparities across intersectional racial, gender, or socioeconomic lines [109].

Underpinning all these efforts are the foundational imperatives of data privacy and cybersecurity. While FL provides a promising cryptographic solution—enabling decentralized model optimization without the direct transfer of sensitive Protected Health Information (PHI)—striking an optimal balance between the data sharing required for algorithmic refinement and the strict preservation of institutional data sovereignty necessitates robust, dynamic governance frameworks [75,110,111]. Advanced computational techniques, such as Data Shapley valuations, which precisely quantify the marginal predictive contribution of individual data sources, can facilitate fair, transparent compensation models for data contributors (i.e., hospitals and patients). This promotes equitable data-sharing consortia while strictly upholding individual privacy rights [112].

As Kelly et al. identify, the urgent adaptation of regulatory science and the codification of unequivocal accountability mechanisms remain the most significant non-technical hurdles to realizing true clinical impact [84]. Therefore, the translational path forward demands an aggressively concerted, multidisciplinary effort uniting clinicians, computational researchers, industry stakeholders, regulatory bodies, and bioethicists. The ultimate objective must be the co-creation of a healthcare ecosystem that simultaneously fosters rapid technological innovation while uncompromisingly prioritizing patient safety. This requires rigorous oversight, continuous post-market algorithmic auditing to detect and rectify “model drift,” and the establishment of transparent, enforceable ethical guidelines. Only through the execution of such a holistic, ethically grounded approach can the profound clinical potential of artificial intelligence be responsibly and safely realized in the management of patients with intracerebral hemorrhage.

### 4.6. Evidence Gap and Translation Chasm

The challenges enumerated above collectively contribute to a persistent “translation chasm” between AI research and clinical practice, maintained not only by technical barriers but by fundamental evidence gaps.

External validation studies remain scarce. When models developed on single-institution data are evaluated across different populations or acquisition protocols, performance degradation of 10–20% is common, undermining clinical confidence. This vulnerability exposes a critical lack of out-of-distribution robustness. Without rigorous, large-scale external validation across heterogeneous clinical environments, the reproducibility of these models remains unproven, rendering them unsafe for general clinical deployment. Prospective validation is exceptionally rare, and adherence to reporting guidelines such as TRIPOD-AI or CONSORT-AI remains inconsistent, raising concerns about selective reporting and irreproducible performance metrics.

Comparative effectiveness research is largely absent. Technical metrics like Dice coefficient or AUC are not substitutes for demonstrating superiority against existing clinical standards—whether manual segmentation, clinical risk scores, or conventional surgical planning. Showing improved prediction is insufficient without evidence that such improvement leads to earlier interventions or better outcomes.

Sample size inadequacy further complicates interpretation. Many studies are statistically underpowered, and the “one-variable-per-ten-events” heuristic is frequently violated, leading to overfitting and optimistic performance estimates. Addressing this scaling limitation requires a concerted shift toward building massive, multi-institutional data consortia that provide the statistical power necessary for training and externally validating truly generalizable models. For BCI and emerging technologies, current studies lack long-term follow-up, adequate control conditions, and blinded outcome assessments necessary to distinguish treatment effects from placebo or spontaneous recovery.

Addressing these gaps requires a paradigm shift: moving beyond technical innovation as an endpoint and embracing the rigorous methodologies of clinical research—prospective design, intention-to-treat analysis, blinding, and registered outcomes.

**Figure 2:**
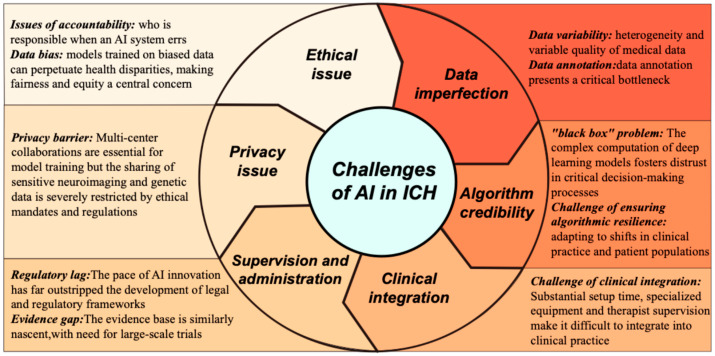
The deficiency of AI in ICH.

Figure 2 illustrates the challenges of Al in ICH from data imperfection, algorithm credibility, clinical integration, supervision and administration, privacy issue, ethical issue.

## 5. Navigating the Future of AI in ICH

The preceding sections have charted both the remarkable advances and the persistent challenges in applying AI to ICH care. From automated segmentation to brain–computer interfaces, the technological landscape is rich with innovation. Yet the translation chasm remains wide: exploratory studies vastly outnumber confirmatory evidence, and clinical adoption lags far behind technical capability. Navigating the future requires not merely continued innovation, but a fundamental reorientation toward rigorous validation, seamless integration, and patient-centered impact. The following directions represent not exhaustive possibilities, but priority areas where focused effort could accelerate meaningful translation.

This review systematically examines recent advancements in the application of AI for the diagnosis and management of ICH, delineating a transformative paradigm shift from basic automated diagnostics toward precision, personalized intervention. Our synthesis indicates that AI architectures, particularly deep learning models, have achieved performance metrics commensurate with, or even exceeding, those of human experts in critical tasks. These include the automated volumetric segmentation of hematomas and PHE [113], as well as the robust prediction of hematoma expansion [114] and long-term functional outcomes. Crucially, the utility of AI now extends far beyond isolated image analysis to encompass the entire clinical trajectory. It is fundamentally reshaping surgical precision via augmented reality (AR) navigation platforms, pioneering novel therapeutic avenues for neural functional restoration through brain–computer interfaces (BCIs), and engineering increasingly sophisticated prognostic models via the integration of high-dimensional, multimodal datasets [115]. Collectively, these advancements herald a future wherein AI is inextricably embedded within the ICH care continuum, catalyzing the transition from standardized, population-level protocols toward highly individualized precision medicine.

Notwithstanding these promising prospects, the translation of AI from in silico research prototypes into robust, bedside clinical tools remains fraught with significant systemic challenges. Throughout this review, we have critically evaluated the pervasive limitations currently impeding translational progress.

The architectural development of the vast majority of contemporary AI models relies disproportionately on non-contrast CT as a singular data modality. However, the pathophysiology of ICH is profoundly complex, characterized by dynamic cascades of neuroinflammation, progressive edema, and secondary brain injury. Consequently, precise clinical management and prognostic stratification urgently necessitate the algorithmic integration of multidimensional data streams, encompassing perfusion neuroimaging, comprehensive laboratory biomarkers, genomic signatures, and continuous, real-time physiological telemetry. The current translational reality is constrained by a conspicuous dearth of standardized, large-scale, multi-institutional ICH registries. This is compounded by inconsistent data fidelity and profound semantic heterogeneity across disparate hospital informatics systems (e.g., PACS, EHRs, and LIS). These structural deficiencies directly precipitate a “generalization gap” (or domain shift), wherein an algorithm exhibiting superlative performance at the training institution suffers catastrophic degradation when deployed in a novel clinical environment [84].

Furthermore, the relatively shallow integration of foundational biomedical research into ICH-specific AI architectures constitutes a formidable bottleneck. At its core, clinical practice is intrinsically interventional; it seeks to resolve causal counterfactuals, such as predicting how an individual patient’s trajectory would alter under specific therapeutic regimens. While current AI models exhibit extraordinary proficiency in mapping complex statistical patterns within imaging and clinical arrays, their structural comprehension of the underlying molecular mechanisms, cellular interactions, and definitive pathophysiological causal chains remains fundamentally limited. This paradigm of “predictive accuracy without mechanistic understanding” severely compromises model interpretability and extrapolative robustness [116]. Plagued by this inherent “black-box” opacity, most models fail to garner clinical trust and are mathematically incapable of executing rigorous counterfactual reasoning. Consequently, future research must aggressively pivot toward a Causal AI paradigm. The integration of formal causal inference frameworks and causal representation learning is imperative to equip models with the capacity to isolate features possessing stable, mechanistic relationships with clinical end-points, thereby filtering out spurious correlational noise [117].

To transcend these pervasive limitations, we propose that future research prioritize the following strategic directions:

Developing Multimodal Medical Agents: The ultimate translational vision is the engineering of multimodal medical agents—sophisticated AI entities capable of natively ingesting and synthesizing diverse clinical inputs, including unstructured clinical narratives, multidimensional neuroimaging, continuous waveform telemetry, and natural language dialogue. These agents will function as high-throughput clinical co-pilots, autonomously managing data integration, executing preliminary risk stratification, and streamlining clinical documentation. Extending this paradigm, the implementation of “multi-agent debate” architectures could facilitate algorithmic consultations among domain-specific AIs (e.g., a specialized neuroimaging AI conferring with a distinct pathophysiological prognostic AI). This dialectical synthesis of computational perspectives would yield exceptionally comprehensive and robust decision support for complex, refractory cases [115].

Engineering Dynamic Intervention Systems: Future AI architectures must evolve from static, cross-sectional predictive tools into dynamic, continuously learning systems. Leveraging frameworks such as Continual Learning, these systems will autonomously recalibrate prognostic assessments and dynamically optimize personalized interventional regimens in direct response to the real-time physiological evolution of the patient (e.g., longitudinal monitoring via low-field portable MRI). This establishes a responsive, closed-loop clinical pathway: continuous assessment, targeted intervention, and algorithmic reassessment.

Pioneering Digital Twins and Simulation-Based Reasoning: The application of “digital twin” technology—the computational generation of high-fidelity, highly parameterized virtual in silico models of individual patients—empowers clinicians to prospectively simulate the physiological consequences of divergent therapeutic strategies within a risk-free computational sandbox. This facilitates rigorous, simulation-based counterfactual reasoning, thereby elevating the prognostic and interventional utility of AI to an unprecedented clinical standard [118].

Achieving Holistic Clinical Integration: Translational research must pivot from the proliferation of isolated, single-task algorithms toward deep, synergistic integration. The overarching objective is the construction of a cohesive, closed-loop clinical ecosystem that seamlessly interweaves automated diagnostics, dynamic prognostication, and precision interventional guidance.

The iterative velocity of AI development exponentially outpaces the lifecycle of traditional medical hardware. Yet, the inviolable cornerstone of modern medicine remains rigorous, evidence-based practice. Currently, high-level clinical evidence validating the efficacy of AI in ICH management—specifically, adequately powered, prospective, multi-center randomized controlled trials (RCTs)—is acutely scarce [119]. The field currently navigates a profound translational tension: must we indefinitely delay clinical implementation while awaiting protracted, gold-standard validation for every algorithmic iteration, or do we accept the inherent risks of deploying incompletely validated technologies to expedite potential patient benefit? This represents a fundamental scientific, ethical, and regulatory impasse.

Consequently, the architectural design of novel, pragmatic clinical trial platforms capable of rapidly and safely evaluating AI utility is an urgent imperative. Furthermore, the establishment of dynamic regulatory frameworks and rigorous post-market surveillance protocols, explicitly tailored to the continuously evolving nature of algorithmic models, is essential to ensure the sustainable and safe maturation of this field. This urgency underscores a pervasive translational limitation: the in silico development of numerous AI tools remains fundamentally divorced from the reality of acute clinical workflows.

Irrespective of superlative technical metrics, an algorithm’s clinical utility is functionally nullified if it cannot be seamlessly embedded into deeply entrenched diagnostic and therapeutic pathways. Successful translational integration strictly mandates a “clinician-in-the-loop” design philosophy. This requires the deep, continuous incorporation of workflow realities, physician requirements, and patient-reported outcomes from the very inception of the algorithmic design process [100]. Overcoming this barrier is not merely a computational challenge; it is a profound systems engineering endeavor aimed at architecting a patient-centric, data-driven, and continuously learning smart healthcare ecosystem. Ultimately, the paramount objective of AI in neurocritical care is not the autonomous displacement of the clinician. Rather, it is to serve as an indispensable computational collaborator—expertly executing data-intensive analytics and high-dimensional pattern recognition. This vital offloading liberates physicians to concentrate exclusively on higher-order cognitive tasks: synthesizing complex clinical gestalts, navigating nuanced ethical dilemmas, and delivering profoundly empathetic, humanistic care, thereby realizing the ultimate paradigm of collaborative medical intelligence [97].

## 6. Discussion

Despite its comprehensive scope, this review is subject to several limitations that should be acknowledged. First, although a systematic search methodology was employed for literature synthesis, no formal quality assessment or risk-of-bias analysis was conducted on the included studies or specific models discussed, which may affect the robustness of the conclusions drawn. Second, foundational research applying AI to investigate ICH pathogenesis or discover biological markers remains scarce, rendering our summary of these aspects necessarily incomplete. Third, the pronounced lack of prospective randomized controlled trials for AI tools in intracerebral hemorrhage—coupled with fundamental computer science challenges such as poor model generalization and high operational costs—forms a major barrier to translational application. This dual deficiency obscures not only the true clinical value but also the cost-effectiveness and practical feasibility of large-scale implementation for many models across diverse healthcare environments. Finally, while our review primarily focuses on the technical and clinical aspects of AI integration and touches upon ethical and regulatory challenges at a societal level, a deeper analysis of socio-technical barriers—including physician acceptance, workflow redesign, and health economic implications—lies beyond the scope of this article, though these factors are undeniably critical for successful and widespread implementation. Beyond these methodological considerations, a critical appraisal of the existing literature must also recognize the substantial heterogeneity in evidentiary maturity across studies. As illustrated in Table 1, AI research in ICH spans a spectrum from exploratory retrospective model-development studies—which remain the most prevalent—to externally validated predictive models, and, in rare instances, prospective clinical evaluations or real-world implementations. This distinction is not merely taxonomic; it carries direct implications for clinical interpretability and translational readiness. Models that have undergone rigorous external validation [31,33,34] warrant greater confidence than those confined to single-center retrospective derivation, while prospective evaluations [32,99]—though still exceptional—provide the most compelling evidence of real-world utility. Future research should explicitly position its contributions along this spectrum and prioritize efforts to advance models from exploratory derivation toward external validation and, ultimately, prospective assessment of clinical impact.

Beyond the immediate context of intracerebral hemorrhage, the AI methodologies surveyed in this review—particularly deep learning-based segmentation, multimodal prognostic modeling, and brain–computer interfaces—carry substantial translational potential for other neurological and oncological conditions. For instance, automated hematoma segmentation frameworks such as U-Net and its variants have been successfully adapted to brain tumor delineation in MRI, while generative augmentation techniques originally developed for oncological imaging are poised to enhance ICH datasets. In real-world clinical settings, the integration of these tools into existing workflows could reduce manual measurement variability, expedite triage decisions, and enable precision monitoring across diverse patient populations. However, successful generalization requires careful validation against domain-specific data heterogeneity, imaging protocols, and outcome definitions. Prospective multicenter studies evaluating the portability of ICH-trained models to other hemorrhagic or space-occupying lesions—and vice versa—will be essential to establish robust, cross-disease AI platforms. Such efforts align with the broader vision of a learning healthcare system, where AI continuously refines its performance across institutions and indications, ultimately improving patient outcomes at scale.

## 7. Conclusions

The integration of artificial intelligence (AI) into the clinical management of intracerebral hemorrhage (ICH) transcends a mere technological upgrade; it heralds a fundamental paradigm shift toward proactive, personalized, and precision neurocritical care. This review has synthesized the rapid evolution of AI applications, from achieving expert-level proficiency in automated neuroimaging analysis to constructing high-fidelity prognostic models and pioneering novel therapeutic modalities via brain–computer interfaces (BCIs). These computational tools possess the undeniable potential to optimize the entire clinical continuum, spanning from hyperacute diagnosis to long-term functional rehabilitation.

Nevertheless, the translational trajectory from algorithmic development to routine bedside deployment remains fraught with formidable systemic barriers. The maturation of promising in silico prototypes into robust clinical instruments is currently impeded by the inherent fragility of models trained on heterogeneous datasets, the pervasive “black-box” opacity that undermines clinical trust, and the profound ethical and regulatory dilemmas precipitated by increasingly autonomous systems. Successfully navigating this translational chasm necessitates a concerted, multidisciplinary consensus among data scientists, clinicians, ethicists, and regulatory bodies.

Crucially, the ultimate objective of medical AI is not to supplant the clinician, but to cultivate a framework of “collaborative intelligence.” Within this paradigm, AI executes data-intensive computational workflows, thereby liberating physicians to concentrate on higher-order diagnostic synthesis, complex clinical decision-making, and the indispensable humanistic dimensions of patient care. By embracing this synergistic vision and systematically resolving extant methodological and regulatory bottlenecks with rigorous empirical validation, the biomedical community can fully leverage AI to redefine the standard of care and substantively improve clinical outcomes for patients afflicted by ICH.

## Figures and Tables

**Table 1:** Representative AI Studies in ICH Categorized by Evidentiary Maturity.

Exploratory Retrospective Model-Development Studies	Externally Validated Predictive Models	Prospective Clinical Evaluations or Real-World Implementations
Uncertainty-aware deep learning framework for perihematomal edema segmentation [14]	ICH-LR2S2 risk score for stroke-associated pneumonia [31]	Low-field portable MRI with deep learning reconstruction for ICH detection [32]
3D U-Net for automatic segmentation of intraparenchymal and intraventricular hemorrhage [15]	H-SYSTEM knowledge graph-enhanced model for hypertensive ICH [33]	Centralized triage algorithm within a distributive stroke network [99]
Robust deep learning segmentation method for hematoma volumetric analysis [27]	ICH-PRNet cross-modal prognostic prediction model using joint-attention interaction [34]	Prospective randomized trials remain scarce; real-world implementation evidence is currently limited to early-stage clinical integration studies

## Data Availability

No new data were created or analyzed in this study. Data sharing is not applicable to this article.
